# Odorant-binding proteins (OBPs) in *Ceratitis capitata* Wiedemann (Diptera: Tephritidae): integrative analysis of a multigene family

**DOI:** 10.1093/jisesa/ieaf088

**Published:** 2025-10-21

**Authors:** Brenda Torres-Huerta, Obdulia L Segura-Leon, José S Meza, Juan Cibrián-Tovar, Guadalupe Reyes-Santiago, Lauro Soto-Rojas

**Affiliations:** Fitosanidad Entomología y Acarología, Colegio de Postgraduados Campus Montecillo, Texcoco de Mora, State of Mexico, 56264, Mexico; Fitosanidad Entomología y Acarología, Colegio de Postgraduados Campus Montecillo, Texcoco de Mora, State of Mexico, 56264, Mexico; Departamento de Genética, Programa Operativo de Moscas, SADER-SENASICA/IICA, Metapa de Dominguez, Chiapas, 30860, Mexico; Fitosanidad Entomología y Acarología, Colegio de Postgraduados Campus Montecillo, Texcoco de Mora, State of Mexico, 56264, Mexico; Parasitología Agrícola, Universidad Autónoma de Chapingo, Texcoco de Mora, State of Mexico, 56230, Mexico; Fitosanidad Entomología y Acarología, Colegio de Postgraduados Campus Montecillo, Texcoco de Mora, State of Mexico, 56264, Mexico

**Keywords:** pest, transcriptome, genome, chemosensory proteins, molecular evolution

## Abstract

The Mediterranean fruit fly (*Ceratitis capitata* Wiedemann, medfly) is a highly invasive agricultural pest with a considerable threat to global fruit production. Its olfactory system, mediated by odorant-binding proteins (OBPs), plays a fundamental role in key behaviors such as host localization, mate recognition, and oviposition site selection. This study presents a comprehensive homologation and systematic reclassification of CcapOBPs through comparative genomic analyses with the model organism *Drosophila melanogaster* Meigen and 8 Tephritidae species (tribes Dacini, Toxotrypanini, and Trypetini), supported by Bayesian phylogenetic inference. By integrating 2 genome assemblies (Ccap_2.1 and EGII-3.2.1), additional GenBank entries, a de novo head transcriptome from sexually mature wild males, and comparative analyses with *D. melanogaster* orthologs, we consolidated 156 candidate sequences into a homologized repertoire of 48 CcapOBPs. RT-PCR validation of 21 representative Ccap*Obp*s confirmed their expression in male heads and highlighted how the de novo transcriptome recovered genes missing from individual genome assemblies, demonstrating consistency across all data sources. Phylogenetic reconstruction of CcapOBP and tephritid species revealed clustering patterns consistent with the established evolutionary relationships within the family, enabling the identification of ortholog genes, lineage-specific diversification events, gene duplications, expansions in *C. capitata*. However, limitations were identified in datasets for the other fruit fly species, and the need for nomenclature adjustments based on chromosomal localization. This study represents the most comprehensive OBP homologation in medfly to date, providing a robust framework for understanding the molecular evolution of chemosensory systems in Tephritidae and supporting the development of species-specific and environmentally sustainable pest management strategies.

## Introduction

Modern agriculture faces numerous challenges, including developing species-specific and environmentally sustainable pest management strategies that reduce the use of insecticides (Tudi et al. 2021). An emerging approach involves the molecular study of the biological components underlying insect communication systems (Lizana et al. 2022). The insect olfactory system consists of chemosensory proteins that participate in perireceptor processes and facilitate the transport of odorant molecules from the environment through the sensillar lymph to transmembrane receptors on olfactory neurons for activation. This process converts chemical signals into electrical signals, triggering specific physiological and behavioral responses in insects (Sachse and Krieger 2011, Leal 2013).

The first step in insect olfactory processing is mediated by odorant-binding proteins (OBPs). These globular proteins, exclusive to insects and with no homology to vertebrate binding proteins, function as the primary filter in recognition of info chemicals that regulate vital behaviors such as host location, mating, oviposition site selection, and defense (Zhou et al. 2010, Zhu et al. 2019). While OBP research has provided fundamental insights into insect olfactory perception, studies on economically important species have significantly increased over the past 15 years, as understanding these proteins lays the basis for developing innovative tools that can be integrated into pest management strategies (Venthur and Zhou 2018).

Among the biotechnological applications in crop protection, reverse chemical ecology has emerged as a key approach for identifying novel, behaviorally active compounds. This approach employs protein modeling and molecular docking to screen candidates for interactions with chemosensory proteins (P D et al. 2014, Rana et al. 2024). Additional applications include the development of multipurpose biosensors and the modulation of insect behaviors, such as host colonization and mating, through gene silencing or genetic editing (Pelosi et al. 2018, Brito et al. 2020). Furthermore, due to their small molecular size, OBPs can be efficiently expressed in bacterial and yeast expression systems, facilitating purification via chromatography. Their three-dimensional structure provides high stability against solvents, elevated temperatures, and proteolytic degradation, making them optimal candidates for biotechnological applications (Lartigue et al. 2004, Pelosi et al. 2014, Brito et al. 2020).

Advances in omics technologies, bioinformatics, and computational biology have greatly facilitated the identification and characterization of OBP repertoires in non-model insects, particularly in economically important species such as tephritid fruit flies (Ono et al. 2021). Among them, the Mediterranean fruit fly (*Ceratitis capitata* Wiedemann), or medfly, is considered one of the most globally invasive pests. The FAO/International Atomic Energy Agency (IAEA) Centre of Nuclear Techniques in Food and Agriculture classifies it as a major threat to global agricultural production (IAEA, n.d.). In Mexico, the medfly is designated a quarantine exotic pest (Aluja and Mangan 2008). Although the country has been officially declared medfly-free, its presence in Central and South America, combined with high trade volumes, poses a constant risk of introduction into southeastern Mexico and other regions (Guzmán-Plazola 2010, Liedo 2024).

Despite the economic importance of the medfly, research on its OBPs remains limited and ambiguous. To date, only 4 studies have addressed OBP identification and characterization. Siciliano et al. (2014a) identified 17 CcapOBPs from 3 EST libraries derived from different tissues, describing their tissue-specific distribution in the olfactory organs of 4-day-old males and females using RT-PCR. They also examined the expression of 5 CcapOBPs (*obp19d-1*, *obp69a*, *obp83a-1*, *obp83a-2*, and *obp28a*) in relation to sexual maturity and mating. Additionally, they analyzed the binding affinity of 15 male pheromone compounds, highlighting the specificity of CcapOBP83a-2 for (E, E)-α-farnesene, a major component of the male pheromone of *C. capitata* (Siciliano et al. 2014b).

Subsequently, Papanicolaou et al. (2016) reported 46 genes in the Ccap_2.1 (AOHK02) genome assembly. However, their analysis lacked detailed classification and assigned numerical identifiers consecutively, which introduced ambiguities in later studies. For example, Falchetto et al. (2019) referred to CcapOBP83a-2 as OBP24 and studied OBP22, previously identified as OBP69a. The latter was shown to be transcribed in the antennae and maxillary palps, with affinity for several terpenoid compounds, including (E, E)-α-farnesene, suggesting a role in intersex olfactory communication.

The lack of precise homologation limits comparative analyses and the effective use of genetic and functional data from other tephritid species. This issue complicates data integration and slows advancements in understanding olfactory mechanisms. Many OBPs remain insufficiently characterized or improperly homologated relative to well-studied models like *D. melanogaster*, leading to nomenclature inconsistencies and hindering the extrapolation of functional and evolutionary insights. Thus, establishing homologous relationships between CcapOBPs and reference organisms is essential to resolve these limitations and support a more integrated understanding of OBP function and evolution.

This study consolidated the OBP repertoire of *C. capitata* through an integrative analysis combining 2 available genome assemblies (Ccap_2.1 [AOHK02] and EGII-3.2.1 [CAJHJT01]), additional GenBank entries, and a de novo transcriptome from the heads of sexually mature wild males (F_1_), a tissue selected for its central role in olfactory signal detection. Homologation and reclassification were guided by comparative genomic data from *D. melanogaster*, and diversification within Tephritidae species was evaluated using Bayesian phylogenetic inference. We validated 21 OBPs via RT-PCR, selected based on their expression in chemosensory tissues of *D. melanogaster* and their detection patterns across data sources. This work represents the most comprehensive OBP homologation in *C. capitata* to date and provides a robust framework for future functional and comparative studies in Tephritidae.

## Materials and Methods

### De Novo Transcriptome Assembly and OBP Mining

We collected *C. capitata*-infested coffee fruits in Chimaltenango, Guatemala, in collaboration with the Moscamed program (SENASICA). Fruits were placed in containers for larval emergence, and larvae were then transferred to sawdust-filled containers for pupation. After 13 ± 1 days, adults emerged in plexiglass cages and were fed a diet consisting of a 3:1 sugar-to-protein mixture (Yeast Hydrolysate Enzymatic, BP Biomedicals, LLC). After 7 to 10 days, mated females were provided with guavas (*Psidium guajava* L.) for oviposition and egg collection to obtain the F_1_ generation, following the same protocol as for the parental strain. Emerging F_1_ adults were sexed, and males were maintained in cages under a 12:12 h light:dark cycle and fed the same diet until reaching sexual maturity.

We collected 150 sexually mature males (7 to 10 days) during the calling stage (07:00 to 09:00 h) and preserved them in RNAlater, storing them at −20  °C. Adult heads were dissected and pooled into 3 groups of 20 heads each. Head tissue was chosen due to its central role in sensory integration and olfactory signal processing, as well as to complement genome assemblies by capturing OBPs actively expressed in sexually mature F_1_ males from wild populations. Samples were sent to BGI (Hong Kong, China) for RNA extraction and quality assessment using an Agilent 2100 Bioanalyzer. Six paired-end libraries were prepared using the MGIEasy RNA Library Prep Kit (MGI-Tech), and sequencing was performed on the BGISEQ-500 platform, generating 100 bp reads and a total of 4 Gb of data.

FastQC v0.10.1 (Babraham Institute, Cambridge) was used to assess read quality, and FastP (Chen et al. 2018) removed low-quality reads (<Q30) and adapter sequences. We performed the de novo assembly with Trinity v2.06 (kmer = 25) (Haas et al. 2013), followed by quality evaluation using QUAST v.0.3.0 (Gurevich et al. 2013). TransDecoder v.5.5.0 (Haas, BJ. https://github.com/TransDecoder/TransDecoder) predicted open reading frames (ORFs) and coding regions, setting a minimum length of 100 amino acids. Functional annotation of the resulting protein sequences was conducted through conserved domain identification with InterProScan (Jones et al. 2014). Finally, sequences containing domains from the insect pheromone/OBP superfamily (SSF47565) and the PBP/GOBP family (PF01395) were extracted.

### Mining of CcapOBP Sequences from National Center for Biotechnology Information and Reported Genomes

Protein sequences of *C. capitata* were retrieved from the non-redundant protein database at the National Center for Biotechnology Information (NCBI), including those encoded by the Ccap_2.1 (AOHK02) and EGII-3.2.1 (CAJHJT01) genome assemblies. The retrieved sequences were compiled into a single dataset and assigned identifiers based on their source database. Conserved domain analysis was performed using InterProScan, extracting sequences annotated within the insect pheromone/OBP superfamily (SSF47565) and the PBP/GOBP family (PF01395).

### Classification and Comparative Analysis of CcapOBPs with *D. melanogaster*

Protein sequences obtained from the wild male transcriptome and the NCBI database, including those from the Ccap_2.1 and EGII-3.2.1 genomes, were compiled into a single dataset after matching the PBP/GOBP family and the OBP superfamily. Homology analyses were performed using BLAST+ (Camacho et al. 2009) against a local database built from *D. melanogaster* OBPs reported in FlyBase.

We conducted a Bayesian phylogenetic analysis to assess the evolutionary relationships of *C. capitata* OBPs. The dataset included CcapOBP sequences from the transcriptome and NCBI and *D. melanogaster* OBPs previously used in the homology analysis. Sequences were aligned using the iterative refinement method in MAFFT (Katoh et al. 2002) and visualized in AliView (Larsson 2014). The best evolutionary model was selected with ModelTest-NG under the Bayesian Information Criterion (BIC) (Darriba et al. 2020). Phylogenetic reconstruction was performed in BEAST v 2.7.8 (Suchard et al. 2018), running 20 million generations. Convergence and stability of Markov Chain Monte Carlo (MCMC) chains were evaluated in Tracer v1.7.1, discarding 30% of initial trees as burn-in. The final phylogenetic tree was edited using iTOL v6.5.8 (Letunic and Bork 2024).

Following homology analyses and phylogenetic reconstruction, redundant sequences were grouped and renamed using the prefix Ccap, followed by the corresponding OBP name based on the best BLASTp match and phylogenetic clustering. A representative sequence was selected for each CcapOBP, prioritizing the complete sequence, and the ORFs were verified using ORFinder (Rombel et al. 2002). All CcapOBP sequences were homologated, generating a reference dataset with ORFs and structural features. Based on these reference sequences, CcapOBPs were classified according to conserved cysteine profiles. SignalP-5.0 (DTU Health Tech, Lyngby, Denmark) was also used to predict the presence of signal peptides.

### RT-PCR Validation of Selected CcapOBPs

From the curated CcapOBPs, we selected 21 genes for RT-PCR validation based on 2 complementary criteria: orthology to *D. melanogaster* OBPs that are expressed in chemosensory tissues (arista, antenna, proboscis, tarsi, pharynx, eyes) as documented in FlyBase and previous studies (Galindo and Smith 2001, Larter et al. 2016); and source coverage, ensuring representation of OBPs detected across the different datasets (Ccap_2.1, EGII-3.2.1, GenBank and the transcriptome). Specific primers were designed from the ORFs in Geneious (Kearse et al. 2012) and evaluated with OligoAnalyzer Tool (Integrated DNA Technologies) and Oligo Evaluator (Sigma Aldrich) to ensure melting temperature differences <4 °C. Primers were synthesized at T4OLIGO (Guanajuato, Mexico).

Total RNA was extracted from heads of sexually mature wild-derived F1 *C. capitata* males (7 to 8 days old) with the SV Total RNA Isolation System (PROMEGA) following the manufacturer’s instructions. RNA integrity and quantity were assessed via 2% agarose gel electrophoresis and measured with a Nanodrop 2000 spectrophotometer (Nanodrop Technologies). We synthesized cDNA using 500 ng of total RNA and the GoScript Reverse Transcription Mix, Oligo(dT) (PROMEGA). Gene validation was performed via RT-PCR using GoTaq Colorless Master Mix (PROMEGA) and the previously designed and synthesized primers (Supplementary Table S1).

Amplification was conducted in an Arktik Thermal Cycler (Thermo Scientific). The amplification cycle began with an initial denaturation at 94 °C for 3 min, followed by 30 cycles of 94 °C for 30 s, annealing at a gene-specific temperature for 30 s, and extension at 72 °C for 2 min, concluding with a final extension at 72 °C for 10 min. PCR products were run on a 2% agarose gel stained with Red Stain Gel (Biotium) and imaged with a Quantum ST5 system (Vilber Lourmat). Sequencing was performed by Macrogen Inc. (Seoul, South Korea). We processed the sequences in BioEdit^®^ 7.2 for cleaning and assembly.

### Phylogenetic Analysis of OBPs in Tephritidae Species

We retrieved from NCBI the OBP sequences reported in 8 Tephritidae species representing the tribes Dacini (*Bactrocera dorsalis* Hendel, *Bactrocera oleae* Rossi, *Zeugodacus cucurbitae* Coquillett, *Zeugodacus tau* Walker), Toxotrypanini (*Anastrepha ludens* Loew, *Anastrepha obliqua* Macquart, *Anastrepha fraterculus* Wiedemann), and Trypetini (*Rhagoletis pomonella* Walsh) as listed in Supplementary Table S2. These sequences were analyzed together with our homologized and classified CcapOBPs. A multiple sequence alignment was performed using the iterative refinement method in MAFFT (Katoh et al. 2002) and visualized in AliView (Larsson 2014). The optimal evolutionary model was determined using ModelTest-NG under the BIC (Darriba et al. 2020). Phylogenetic analysis was conducted in BEAST v 2.7.8 (Suchard et al. 2018), set to run for 40 million generations. MCMC output was assessed in Tracer v1.7.1, discarding the first 30% of trees as burn-in. The final phylogenetic tree was edited in iTOL v6.5.8 (Letunic and Bork 2024).

## Results

### De Novo Transcriptome of Wild Males and CcapOBP Mining

BGISEQ-500 sequencing of 6 paired-end libraries generated 559,580,793 raw reads, with an average of 48.66 million reads per library and a mean length of 100 bp. After filtering low-quality reads, each paired-end library retained an average of 48.35 million reads, maintaining a mean length of 99 bp. Read quality remained high, with over 97% and 90% of reads exceeding Q20 and Q30, respectively, while duplication rates remained low (Supplementary Table S3). De novo assembly of the 12 libraries yielded 333,557 transcripts, with a mean length of 484 bp, a GC content of 34.66%, and an N50 of 910 bp. Redundancy filtering refined the dataset to 216,798 unigenes, with a mean length of 382 bp, a GC content of 35.37%, and an N50 of 1,398 bp.

**Fig. 1.**
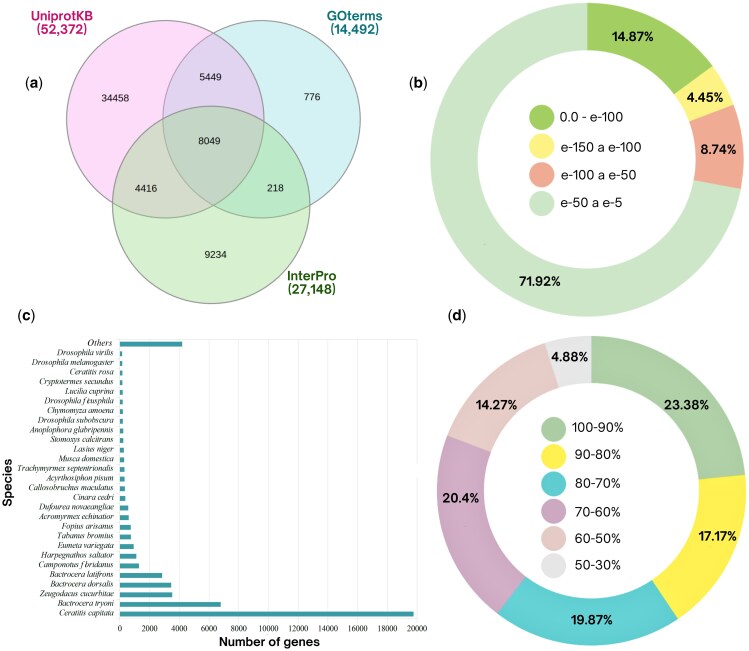
Functional annotation of the de novo head transcriptome from sexually mature wild-derived F_1_ males of *Ceratitis capitata*. a) Venn diagram of 31,065 unigenes annotated with InterPro, Insecta-UniprotKB (BLASTp, E ≤ 1 × 10^−5^), and Gene Ontology (GO); numerals denote overlaps. b) E-value distribution of Insecta-UniprotKB hits. c) Top-hit species distribution of Insecta-UniprotKB hits, bar length = number of unigenes per species. d) Pair-wise amino-acid identity between unigenes and their best UniProtKB match.

As shown in Fig. 1, 67,548 unigenes had annotations in at least 1 database. Blastx searches against the Insecta-UniprotKB database assigned annotations to 77.53% of the unigenes. E-value distribution remained below 1e-50 for 71.9% of the hits, while 80.8% of the unigenes exhibited sequence similarity above 70%, and 23.38% showed 90% to 100% similarity (Fig. 1). Over 90% of *C. capitata* unigenes matched sequences from the Tephritidae family, and more than 70% aligned with sequences from *C. capitata*, *Bactrocera tryoni* Froggatt, *Z. cucurbitae*, and *B. dorsalis* (Fig. 1).

The annotation of 22,029 unigenes from *C. capitata* classified them into 3 Gene Ontology (GO) categories. Among these, molecular function (MF, 38.81%) represented the largest category, followed by biological processes (35.52%) and cellular components (CCs, 25.66%). TransDecoder analysis assigned functional domains to more than 40% of the predicted proteins, which matched entries in InterPro consortium databases (Fig. 1). The analysis identified 29 OBPs (PQ528246 to PQ528274) with complete ORFs (Supplementary Table S4; Table 1). Their *D. melanogaster* orthologs share 25% to 69% amino-acid identity with E-values <1 × 10^−20^. All, except CcapOBP19c, possess an N-terminal signal peptide.

**Table 1.** Results of the BLASTp analysis of *Ceratitis capitata* odorant-binding proteins (CcapOBPs) compared to *Drosophila melanogaster* OBPs

Name	Acces No.	AA	Signal peptide	Clasification	Blastp with DmelOBPs				
					Acc. No.	Description	Evalue	Identity (%)	Coverage (%)
**CcapOBP19a**	XP_004525133	147	1-24	Classical	NP_728338.2	OBP19a	6.04E-60	55.17	98
**CcapOBP19a-like**	PQ528246	147	1-27	Classical	NP_728338.2	OBP19a	2.46E-54	60.00	83
**CcapOBP19b**	PQ528247	151	1-20	Classical	NP_608391.2	OBP19b	8.82E-34	37.58	98
**CcapOBP19b-like**	PQ528248	124	1-25	Classical	NP_523421.2	OBP19d isoform A	1.78E-12	29.63	84
**CcapOBP19c**	PQ528249	175	–	Classical	NP_608392.1	OBP19c isoform A	4.09E-21	25.20	74
**CcapOBP19c-like**	PQ528250	253	1-19	Plus-C	NP_611443.1	OBP56b	0.019634	24.51	65
**CcapOBP19d**	XP_004525035	142	1-21	Classical	NP_523421.2	OBP19d isoform A	7.45E-29	42.73	77
**CcapOBP19d-like**	PQ528251	143	1-21	Classical	NP_523421.2	OBP19d isoform A	6.52E-20	31.30	80
**CcapOBP28a**	PQ528252	147	1-21	Classical	NP_523505.1	OBP28a	1.59E-46	48.55	94
**CcapOBP44a-isoform1**	PQ528253	142	1-18	Classical	NP_610358.1	OBP44a isoform A	1.85E-62	61.54	100
**CcapOBP44a-isoform2**	PQ528254	142	1-18	Classical	NP_610358.1	OBP44a isoform A	1.03E-60	60.14	100
**CcapOBP50e-like**	PQ528255	179	1-22	Plus-C	NP_610959.2	OBP50e	9.86E-12	21.33	76
**CcapOBP47a**	CAD7013140	195	–	Classical	NP_610632.1	OBP47a isoform A	2.20E-31	41.59	57
**CcapOBP47b**	XP_012162233	199	1-29	Plus-C	NP_610669.1	OBP47b	2.43E-68	50.00	97
**CcapOBP49a**	PQ528256	221	1-21	Plus-C	NP_610812.1	OBP49a	4.55E-25	31.93	96
**CcapOBP56b**	XP_004517903	138	1-25	Classical	NP_611443.1	OBP56b	2.74E-23	36.64	90
**CcapOBP56b-like1**	XP_020718148	165	1-25	Classical	NP_611442.1	OBP56a	9.89E-14	28.07	64
**CcapOBP56b-like2**	XP_020718149	138	1-25	Classical	NP_611442.1	OBP56a	8.81E-14	28.07	64
**CcapOBP56c**	XP_012155938	327	–	Classical	NP_725925.3	OBP56c isoform C	1.50E-32	31.90	70
**CcapOBP56d**	PQ528257	137	1-19	Classical	NP_611444.2	OBP56d isoform A	2.78E-34	42.22	99
**CcapOBP56e**	XP_004517904	135	1-20	Classical	NP_611444.2	OBP56d isoform A	1.61E-23	36.22	93
**CcapOBP56g**	PQ528258	124	1-20	Classical	NP_611448.2	OBP56h isoform A	7.76E-23	34.65	95
**CcapOBP56h1**	PQ528259	136	1-20	Classical	NP_611448.2	OBP56h isoform A	6.76E-30	38.40	92
**CcapOBP56h2**	XP_004517905	138	1-20	Classical	NP_611448.2	OBP56h isoform A	9.75E-28	37.88	96
**CcapOBP57c**	PQ528260	186	1-33	Classical	NP_611481.1	OBP57c	4.81E-17	27.56	66
**CcapOBP58c**	XP_004537654	201	1-21	Plus-C	NP_611710.1	OBP58c	1.07E-95	62.56	97
**CcapOBP59a**	PQ528261	342	1-28	Plus-C	NP_788429.1	OBP59a	2.97E-37	60.22	39
**CcapOBP69a**	NP_001295335	147	1-24	Classical	NP_524039.2	OBP69a	1.48E-31	36.84	90
**CcapOBP73a**	PQ528262	102	1-21	Plus-C	NP_001334711.1	OBP73a isoform C	4.50E-90	51.98	95
**CcapOBP83a**	PQ528263	157	1-34	Classical	NP_001287190.1	OBP83a isoform C	3.66E-82	69.43	100
**CcapOBP83a-like**	PQ528264	148	1-24	Classical	NP_524241.1	OBP83a isoform A	3.58E-57	55.17	97
**CcapOBP83cd**	PQ528265	241	1-21	Atypical	NP_649612.1	OBP83cd	3.46E-78	46.08	88
**CcapOBP83ef**	PQ528266	277	1-25	Atypical	NP_731042.1	OBP83ef	1.31E-75	49.06	74
**CcapOBP83g**	PQ528267	142	1-18	Classical	NP_731043.1	OBP83g	1.21E-66	62.14	95
**CcapOBP84a**	PQ528268	178	1-24	Plus-C	NP_476990.1	OBP84a isoform A	4.64E-41	50.00	62
**CcapOBP84a-like**	XP_004529369	177	1-35	Plus-C	NP_476990.1	OBP84a isoform A	3.52E-38	38.73	93
**CcapOBP8a**	PQ528269	162	1-29	Minus-C	NP_727322.1	OBP8a	1.11E-21	32.77	69
**CcapOBP99b**	PQ528270	149	1-18	Classical	NP_651707.1	OBP99a isoform A	2.95E-48	45.78	95
**CcapOBP99c**	PQ528271	101	1-16	Minus-C	NP_651711.1	OBP99c isoform A	1.32E-61	56.84	93
**CcapOBP99c-like1a**	XP_004536902	142	1-16	Minus-C	NP_651711.1	OBP99c isoform A	4.00E-28	39.69	92
**CcapOBP99c-like1b**	XP_023158406	142	1-16	Minus-C	NP_651711.1	OBP99c isoform A	7.00E-57	38.93	92
**CcapOBP99c-like2**	PQ528273	144	1-16	Minus-C	NP_651711.1	OBP99c isoform A	1.94E-31	39.39	92
**CcapOBP99c-like3**	NP_001266316	144	1-16	Minus-C	NP_651711.1	OBP99c isoform A	9.22E-29	40.88	95
**CcapOBP99c-like4a**	XP_004536905	143	1.16	Minus-C	NP_651711.1	OBP99c isoform A	1.00E-17	31.25	89
**CcapOBP99c-like4b**	NP_001295338	143	1-16	Minus-C	NP_651711.1	OBP99c isoform A	4.78E-24	36.84	93
**CcapOBP99c-like5**	PQ528272	144	1-16	Minus-C	NP_651711.1	OBP99c isoform A	2.24E-28	40.30	93
**CcapOBP99d**	PQ528274	150	1-16	Minus-C	NP_651712.1	OBP99d	3.04E-26	42.71	63
**CcapOBPlush**	XP_004522281	152	1-16	Classical	NP_728338.2	OBP19a	1.17E-20	31.29	89

### Mining of CcapOBP Sequences from NCBI and Reported Genomes

To identify *C. capitata* OBPs, we performed a conserved domain annotation using InterProScan. This analysis mined proteins annotated in the PBP/GOBP family and the SSF47565 superfamily, incorporating protein sequences from the NCBI non-redundant database, coding sequences (CDS) from reported genomes, and 28 OBPs identified in the de novo transcriptome of wild male heads. Currently, NCBI records 78,675 nucleotide sequences for *C. capitata*, including 66,484 protein sequences. The Ccap_2.1 (AOHK02) genome contains 22,949 CDS, whereas the EGII-3.2.1 (CAJHJT01) genome includes 24,470 CDS. NCBI lists 63 proteins as CcapOBPs, but only 37 are annotated in the Ccap_2.1 genome, while the EGII-3.2.1 genome lacks CDS or proteins annotated as OBPs.

We conducted a conserved domain analysis on 66,484 protein sequences reported in NCBI, assigning specific identifiers to distinguish proteins in the analyzed genomes. Among these, 128 protein sequences exhibited characteristic OBP domains, identified using PFAM (PBP/GOBP family, PF01395) and SUPERFAMILY (pheromone/general OBP, SSF47565) databases. Additionally, these sequences were annotated with GO terms related to MF in odorant binding (GO:0005549) and the CC of the extracellular region (GO:0005576). Within the Ccap_2.1 genome, we identified 48 OBPs annotated in PFAM and SUPERFAMILY, except for XP_012155811.1 and XP_012156332.1, which appear exclusively in SUPERFAMILY (Supplementary Table S5). In the EGII-3.2.1 genome, we found 33 proteins with OBP domains, including CAD7004525.1 and CAD7013698.1, which are exclusively in SUPERFAMILY (Supplementary Table S6).

### Classification and Comparative Analysis with DmelOBPs

**Fig. 2.**
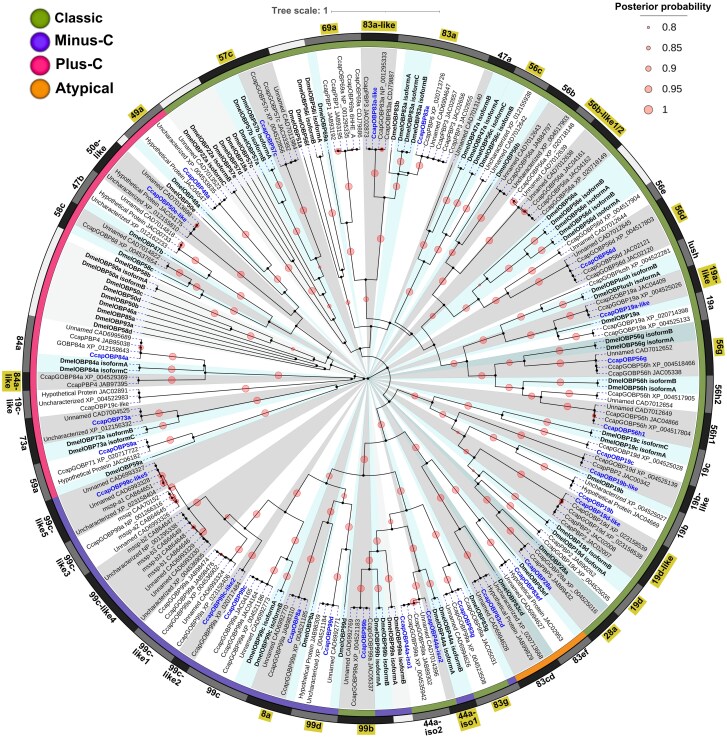
Bayesian phylogeny of *Ceratitis capitata* and *Drosophila melanogaster* odorant-binding proteins (OBPs). Forty-eight CcapOBPs and 73 DmelOBPs (FlyBase) were aligned with MAFFT and analyzed in BEAST v2.7.8 under the WAG + G4 + I model for 20 million MCMC generations. Branch colors denote OBP subfamilies: Classical, Plus-C, Minus-C, and Atypical. CcapOBPs detected in the male-head transcriptome are in navy boldface, and their *D. melanogaster* orthologues are shaded blue. Light and dark-gray outer bands and radial shading delineate the principal OBP clusters. Posterior probabilities (PP ≥ 0.80) are represented by proportional circles. OBPs validated by RT-PCR are highlighted with yellow-shaded labels.

We conducted a phylogenetic analysis using Bayesian inference to compare 73 OBP sequences from *D. melanogaster* (retrieved from FlyBase) with 158 OBP sequences from *C. capitata*, including 129 from NCBI and 29 identified in the de novo transcriptome of wild male heads. The phylogenetic analysis used the WAG + G4 + I model with 20 million generations to ensure robust results. The resulting phylogenetic tree grouped the 158 *C. capitata* sequences into 48 CcapOBPs with posterior probabilities (PP) >90% (Table 1). This analysis unified the classification of CcapOBPs with their orthologous proteins in *D. melanogaster* (Supplementary Table S7) and identified species-specific diversification events in *C. capitata* (Fig. 2).

**Fig. 3.**
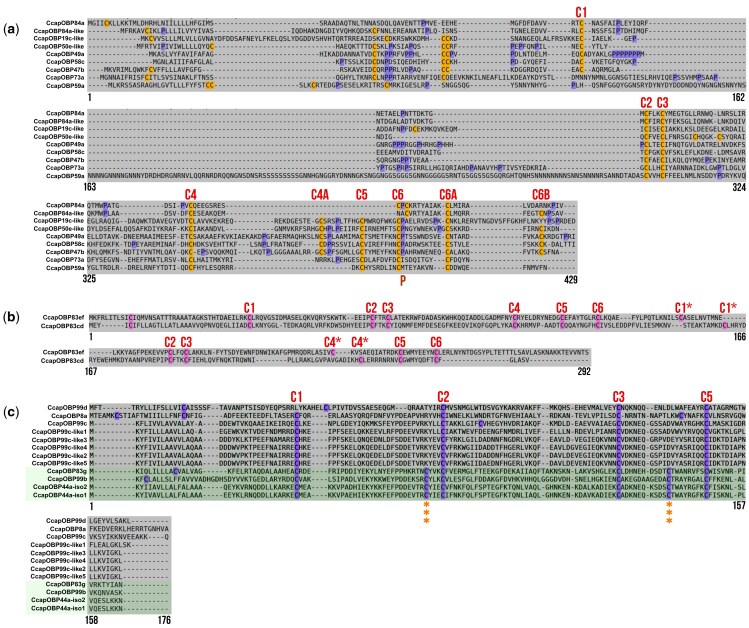
Signature cysteine patterns of non-classical *Ceratitis capitata* OBPs. Amino-acid sequences were aligned with MAFFT and visualized in SeaView v5.0.5 a) Plus-C subfamily, characterized by the presence of more than 6 conserved cysteine residues and a proline. b) Atypical subfamily, which exhibits 2 classical domains; cysteines of the first and second domains are labeled C1 to C6 and C1* to C6*, respectively. c) Minus-C subfamily, with a reduced number of cysteine residues compared to classic OBPs. The green block contains CcapOBPs whose *D. melanogaster* orthologs are annotated as Minus-C but here retain all 6 cysteines (marked with asterisks), displaying a classical pattern.

The phylogenetic analysis grouped the sequences into 3 main clades, reflecting a classification based on the number and pattern of conserved cysteines (Fig. 2). These clades correspond to the classical, Minus-C, Plus-C, and atypical OBP subfamilies. The terminal clade of the phylogenetic tree contained Plus-C OBPs, comprising 28 sequences grouped into 9 CcapOBPs. These OBPs contain 10 conserved cysteines, including the 6 classical residues plus 4 additional ones, and a conserved proline after cysteine 6, a feature not present in all Plus-C OBPs (Fig. 3a). Among these Plus-C OBPs, 6 have orthologs in *D. melanogaster* with similarity >50%, except for CcapOBP49a, which shows 31.93% similarity to DmelOBP49a. Additionally, 3 OBPs (CcapOBP19c-like, CcapOBP84a-like, and CcapOBP46a-like) have lower similarity values ranging between 21.33% and 38.73% (Table 1).

**Fig. 4.**
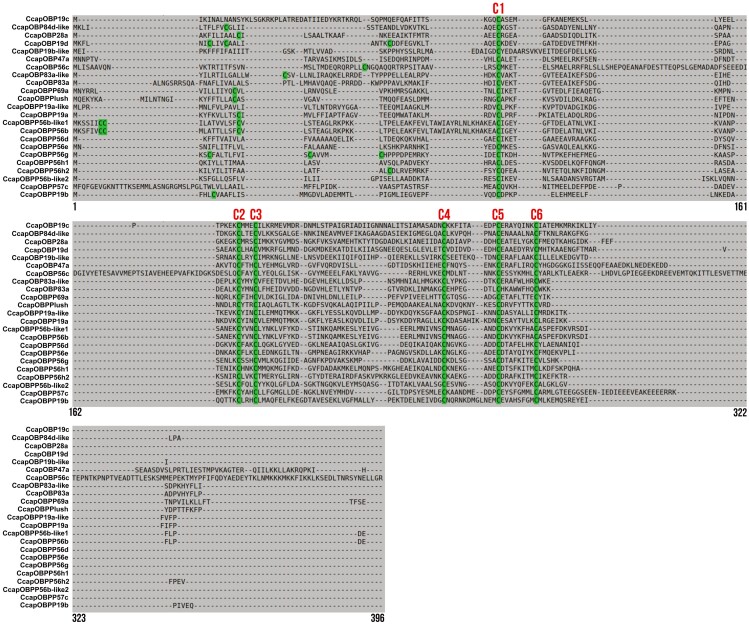
Alignment of the 31 classical odorant-binding proteins (OBPs) from *Ceratitis capitata*. Sequences were aligned with MAFFT and displayed in SeaView v5.0.5. The 6 conserved cysteines (C1 to C6) are highlighted; the hallmark spacings C2-X_3_-C3 and C5-X_8_-C6 are indicated above the alignment. All classical CcapOBPs display the complete six-cysteine motif, distinguishing them from Plus-C, Minus-C, and Atypical subfamilies.

The largest subfamily was the classical OBP, consisting of 72 sequences grouped into 26 CcapOBPs. This subfamily has the characteristic six-cysteine pattern, with some sequences containing additional cysteines in the N-terminal region (Fig. 4). We identified 22 sequences as orthologs of DmelOBPs, with similarity levels ranging from 25% to 69% and E-values below 1.78E-12 (Table 1). Additionally, we identified CcapOBPs exhibiting species-specific diversification in *C. capitata*, as they lack direct orthologs in *D. melanogaster*. In the phylogenetic tree, these sequences cluster within a clade together with CcapOBP orthologs of DmelOBPs, although they do not exhibit a 1-to-1 correspondence (Fig. 2). These OBPs include CcapOBP19a-like, CcapOBP19b-like, CcapOBP19d-like, CcapOBP50e-like, CcapOBP56b-like1-2, CcapOBP83a-like, CcapOBP84a-like, and CcapOBP99c-like1-5.

We identified 2 species-specific duplications in *C. capitata*, designated CcapOBP56h1 and CcapOBP56h2. These duplications are absent in *D. melanogaster* and share 38.41% identity and 63.04% similarity. The phylogenetic analysis divided the classical OBP subfamily into 2 main clades (Fig. 2). The first clade contains 54 sequences from 21 CcapOBPs, all classified as classical OBPs. In contrast, the second clade includes 18 sequences from 6 CcapOBPs, which cluster with atypical and Minus-C OBPs, with PP of 98.7%. Within this second clade, we identified classical OBPs that form a distinct group, consisting of OBP28a, OBP19b, OBP19c, and OBP19d, as well as OBP19b-like and OBP19d-like, which lack homologous orthologs in *D. melanogaster*.

The atypical OBP subfamily is represented exclusively by CcapOBP83cd and CcapOBP83ef, each with 4 orthologous sequences corresponding to DmelOBP83cd and DmelOBP83ef, showing similarities of 46.08% and 49.06%, respectively. This subfamily is characterized by 2 six-cysteine motifs, similar to those found in classical OBPs (Fig. 3b). In the Minus-C subfamily, we identified 37 sequences containing only 4 conserved cysteine and a variable number of residues between them (Fig. 3c). These sequences were grouped into five CcapOBPs: 99d, 8a, 99c, and the 99c-like group. All of these have orthologs in *D. melanogaster*, with similarities ranging from 32% to 62% and E-values below 1.1E-21.

We identified sequences with 6 conserved cysteines, characteristic of classical OBPs, that are related to Minus-C proteins, including CcapOBP83g, CcapOBP44a, and CcapOBP99b (Fig. 3c). These sequences showed similarities >45% with DmelOBP83g, DmelOBP44a, and DmelOBP99b, clustering with PP >99%. Within the Minus-C subfamily, the CcapOBP99c-like group underwent a significant expansion in *C. capitata*. It comprises 24 sequences classified into 5 subgroups (99c-like 1, 2, 3, 4, and 5), with subgroups 99c-like 1 and 4 containing 2 copies (Fig. 2). The CcapOBP99c-like sequences exhibit chromosomal locations indicative of tandem duplication events rather than alternative isoforms (Supplementary Table S7). Despite the absence of direct orthologs in *D. melanogaster*, the 99c-like sequences show similarities >30% with DmelOBP99c isoform A (Table 1).

### RT-PCR Validation of Selected CcapOBPs

Twenty-one CcapOBPs were selected for amplification, based on orthology with *D. melanogaster* OBPs in chemosensory tissues and their representation across data sources (Supplementary Table S1). Among them, 10 CcapOBPs (57c, 49a, 56d, 84a1, 83a, 8a, 56 g, 99 b, 83 g, and 44a-1) were identified in both genomes and the transcriptome. Four CcapOBPs (19a-like, 83a-like, 19d-like, and 28a) were detected exclusively in the Ccap_2.1 genome and the transcriptome, whereas 3 CcapOBPs (84a2, 19d, and 69a) were found only in the Ccap_2.1 genome. Finally, 4 OBPs (56b1, 56c, 56b2, and 99d) were identified in both genomes but not in the transcriptome.

**Fig. 5.**
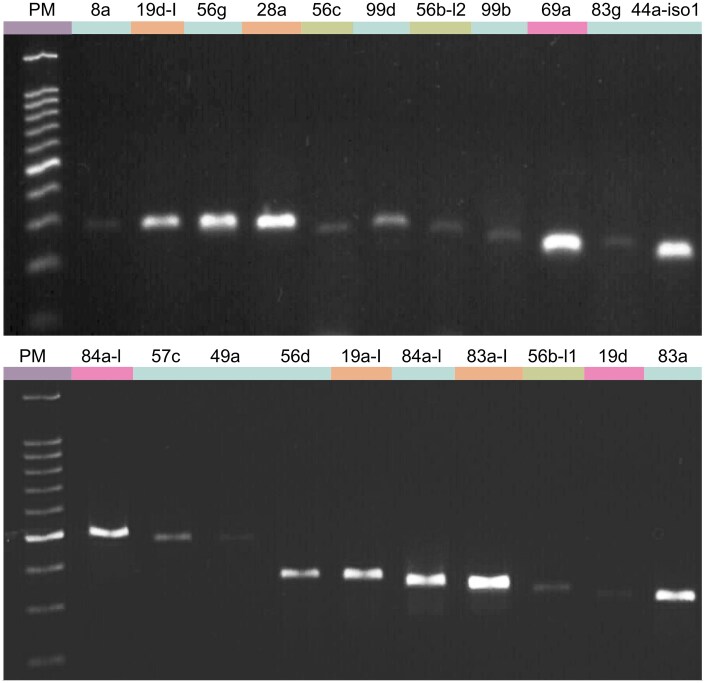
RT-PCR validation of 21 *Ceratitis capitata* OBP transcripts. Amplicons (140 to 220 bp) were resolved on 2% agarose gels. PM, 100-bp size ladder. Colored bars above the lanes denote the dataset(s) in which each OBP was originally detected. cyan: Ccap_2.1 + EGII-3.2.1 + GenBank + male-head transcriptome; orange: Ccap_2.1 + GenBank + transcriptome; green: Ccap_2.1 + EGII-3.2.1; pink: Ccap_2.1 + GenBank. Unique bands of the expected size confirm expression in heads of sexually mature, wild-derived F_1_ males and females (7 to 8 days old).

Despite differences in their detection across the genomes and transcriptome, all selected OBPs were successfully amplified via RT-PCR, confirming their presence and validation (Fig. 5). Amplifications were performed using primer-specific annealing temperatures ranging from 60 °C to 66.4 °C, and the obtained products displayed expected molecular sizes, varying from 206 bp (CcapOBP44a-1) to 480 bp (CcapOBP84a2) (Supplementary Table S1). Additionally, sequencing confirmed their correspondence to the target OBPs, supporting the primer efficiency and their suitability for future studies.

### Phylogenetic Analysis of OBPs in Different Tephritidae Species

**Fig. 6.**
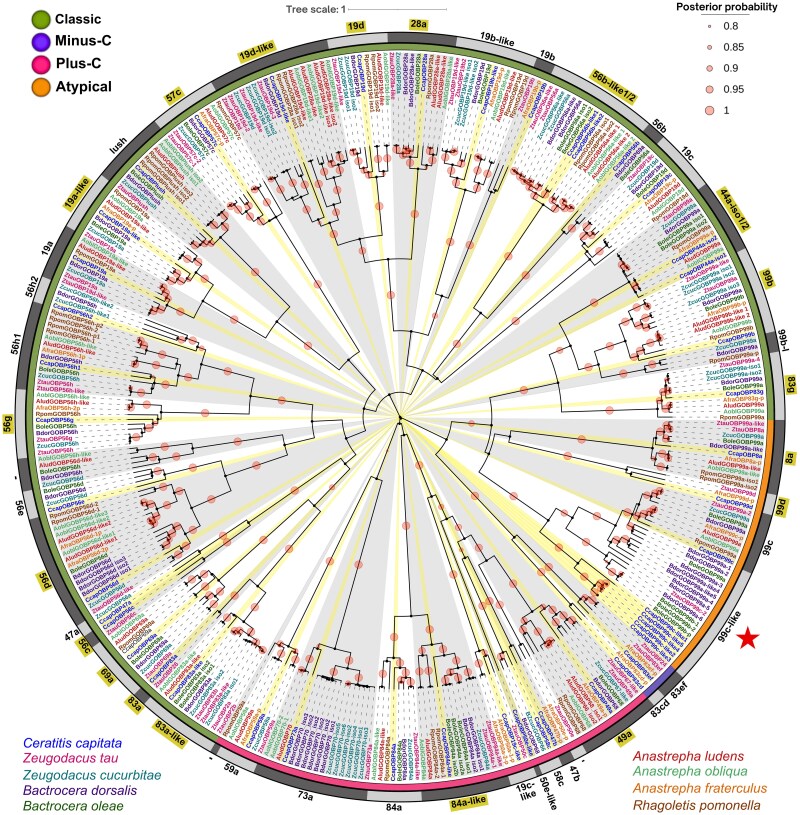
Bayesian phylogeny of Tephritidae odorant-binding proteins (OBPs). Sequences from 9 species (*Ceratitis capitata* [Ccap; 48 OBPs], *Zeugodacus tau* [Ztau], *Z. cucurbita*e [Zcuc], *Bactrocera dorsalis* [Bdor], *B. oleae* [Bole], *Anastrepha fraterculus* [Afra], *A. ludens* [Alud], *A. obliqua* [Aobl], and *Rhagoletis pomonella* [Rpom]) were aligned with MAFFT and analyzed in BEAST v2.7.8 under the JTT + G4 + I model with 40 million of MCMC generations. Branch colors denote OBP subfamilies: Classical, Plus-C, Minus-C, and Atypical. Representatives CcapOBPs that define each cluster are highlighted in yellow and light and dark-gray bars and radial shading group the principal OBP clusters. Posterior probabilities (PP ≥ 0.80) are represented by proportional circles. OBPs validated by RT-PCR are highlighted with yellow-shaded labels. The *C. capitata*-specific expansion of the OBP99c-like group is marked with a star.

We conducted a Bayesian phylogenetic analysis of 307 OBP sequences, including our 48 homologized CcapOBPs and 259 OBPs from 8 Tephritidae species across 3 tribes (Supplementary Table S2). The phylogenetic analysis used the JTT + G4 + I model with 40 million generations to ensure robust results. Based on the number and pattern of conserved cysteines, the sequences clustered into 5 main groups, corresponding to the classical, Plus-C, Minus-C, and atypical OBP subfamilies (Fig. 6). Most OBPs exhibited orthologs in tephritid species, with high phylogenetic support (PP > 90%).

The CcapOBPs exhibited specific clustering patterns with species from different Tephritidae tribes. Most of these proteins formed well-defined clusters with sequences from *Bactrocera* and *Zeugodacus* (tribe Dacini). Additionally, we identified phylogenetic relationships between 8 CcapOBPs and species from the Toxotrypanini (genus *Anastrepha*) and Trypetini (genus *Rhagoletis*) tribes. Some sequences, such as CcapOBP50e-like, did not cluster with reported tephritid proteins but shared over 30% similarity with *D. melanogaster* sequences, retaining characteristic OBP domains and signal peptides.

The atypical OBP subfamilies, represented by CcapOBP83cd and CcapOBP83ef, exhibited limited representation among tephritids, with orthologs identified only in *A. fraterculus* and *Z. tau*. These proteins possess distinct structural features, including 2 six-cysteine motifs, and formed highly supported phylogenetic clusters (PP > 90). Other CcapOBPs, such as 19c-like, 56c, 47a, and 19b, were primarily associated with OBPs reported in *A. fraterculus*, *Z. tau*, and *Z. cucurbitae*. Finally, the CcapOBP99c-like group, characterized by multiple duplications and isoforms, underwent a species-specific expansion in *C. capitata*, similar to that observed in *B. dorsalis*.

## Discussion

The homologation of CcapOBPs is essential in resolving classification and nomenclature inconsistencies in genetic databases. This process enables the precise organization and comparison of available information by leveraging well-studied models such as *D. melanogaster* to infer functional and evolutionary analyses (Hekmat-Scafe et al. 2002, Huang et al. 2016, Venthur and Zhou 2018). These comparisons provide insights into the conservation of this protein family across evolutionarily distant species and help identify species-specific diversification patterns, contributing to a deeper understanding of molecular adaptations in *C. capitata*.

To achieve precise homologation, we integrated available genetic data from NCBI, including the Ccap_2.1 and EGII-3.2.1 genomes, and a de novo transcriptome from heads of sexually mature wild males. RNA-Seq conducted using the BGISEQ-500 platform yielded high-quality data, with 97% of reads achieving a Q20 score, indicating high sequencing accuracy. The de novo assembly produced a robust transcriptome, with 31.15% of unigenes annotated in the Insecta-UniprotKB database. Over 90% of these showed sequence similarity to *C. capitata*, *B. tryoni*, *B. dorsalis*, and *Z. cucurbitae*, indicating strong genetic conservation within the Tephritidae family (Siciliano et al. 2014a, 2014b).

To identify OBPs in the NCBI genomes and transcriptome, we used a domain-based approach targeting conserved protein domains (PF01395/SSF47565) and GO terms associated with odorant binding (GO:0005549) and extracellular region (GO:0005576). This analysis identified 128 OBPs in NCBI, including 48 from the Ccap_2.1 genome and 33 from EGII-3.2.1. Additionally, 29 OBPs were recovered from the de novo transcriptome. These findings demonstrate the effectiveness of combining genomic and transcriptomic mining strategies to characterize complex multigene families, such as OBPs in non-model species (Oppenheim et al. 2015).

Bayesian phylogenetic analysis, based on 128 CcapOBPs from NCBI, 28 from the transcriptome, and 73 *D. melanogaster* OBPs from FlyBase, enabled the homologation of 152 sequences into 48 CcapOBPs. While previous studies reported 65 OBPs in medfly, these lacked robust classification and exhibited nomenclature inconsistencies (Siciliano et al. 2014a, 2014b, Papanicolaou et al. 2016). Issues included redundant naming, inconsistent use of designations such as OBPs, GOBPs, and PBPs, and a high proportion (>50%) of proteins labeled as “hypothetical” or “uncharacterized.” Additionally, several OBPs were misclassified under the male-specific serum polypeptide family, and some, like CcapOBP56d, appeared under multiple identifiers.

The homologation process established a standardized nomenclature based on the phylogenetic relationship between CcapOBPs and DmelOBPs. The “*Obp*” prefix is followed by a cytogenetic region number and a letter denoting gene position (Galindo and Smith 2001, Hekmat-Scafe et al. 2002). This highlights the need for refined OBP annotations in medfly using chromosomal-level assemblies or cytogenetics mapping. For genes lacking annotated 1-to-1 orthologs in *D. melanogaster*, the suffix “-like” was assigned based on robust phylogenetic clustering. For example, CcapOBP83a-like consistently grouped with DmelOBP83a, supporting functional relatedness.

All 48 CcapOBPs retain the conserved domains characteristic of the OBP superfamily, and SignalP predicts an N-terminal signal peptide in 45 of them, as expected for extracellular proteins (Leal 2013). By contrast, CcapOBP19c, CcapOBP47a, and CcapOBP56c lack a detectable signal peptide. Analogous cases have been documented for OBP19c in *A. fraterculus* and *A. obliqua* (Campanini and de Brito 2016), OBP57d in *Drosophila elegans* Bock and Wheeler (Matsuo 2008), and OBP11 in *Spodoptera exigua* Hübner (Zhu et al. 2013). In all instances, the ORFs are complete, and the absence of a predicted signal peptide has been attributed to pronounced N-terminal divergence.

CcapOBPs clustered into classical, Plus-C, Minus-C, and atypical subfamilies, reflecting conserved structural patterns typical of insect OBPs (Pelosi et al. 2014). The classical subfamily was most abundant (26 CcapOBPs), followed by the Plus-C (9), with fewer Minus-C and atypical members, consistent with patterns in other dipterans (Campanini and de Brito 2016, Chen et al. 2019, Segura-León et al. 2022, Wang et al. 2024). Classical OBPs clustered into 2 main clades: 1 exclusively classical and another shared with atypical and Minus-C OBPs, suggesting evolutionary proximity.

Minus-C proteins are conserved across Hymenoptera, Diptera, and Lepidoptera and form a compact clade with well-defined structural features (Lagarde et al. 2011, Spinelli et al. 2012). Structural and phylogenetic data indicate that Minus-C OBPs may be ancestral, with classical OBPs evolving later through the addition of disulfide bridges and increased structural complexity (Spinelli et al. 2012, Sun et al. 2012, Zheng et al. 2016). In honeybees, genomic analyses showed that Minus-C proteins diverged from Classic OBPs by loss or mutation of the second and fifth conserved cysteine residues, supporting structural simplification as their origin in this lineage, although this may not apply to all insects (Mam et al. 2023). Their close relationship is clear, but further comparative studies are needed to determine whether Minus-C OBPs represent ancestral states, repeated simplifications, or convergent ecological adaptations (Zhou et al. 2021).

The clade containing both classical and Minus-C OBPs includes CcapOBP83g, CcapOBP44a1, CcapOBP44a2, and CcapOBP99b, which, although homologous to Minus-C OBPs in *D. melanogaster* (Rondón et al. 2022), display structural features of classical OBPs in multiple sequence alignments. Similarly, as observed in *D. melanogaster*, the Plus-C OBPs in *C. capitata* formed a distinct clade, confirming their monophyletic nature and recent evolutionary divergence (Hekmat-Scafe *et al.* 2002). However, no homologs were identified in the medfly for 9 Plus-C DmelOBPs, highlighting species-specific differences in OBP repertoires.

The analysis revealed species-specific expansions and diversification in CcapOBPs, reflecting selective pressures associated with speciation and ecological adaptations. Although both are dipterans, *D. melanogaster* and *C. capitata* belong to different families and occupy distinct ecological niches (Aluja and Mangan 2008). These differences may have shaped divergent OBP repertoires in *C. capitata*, including duplications such as CcapOBP56h1 and 56h2, expansion of the CcapOBP99c-like group (absent in *D. melanogaster*), and emergence of OBPs without 1-to-1 orthologs, such as CcapOBP83a-like, 19c-like, and 50e-like.

These expansions may have facilitated the specialization of odorant detection, enhancing survival and reproduction in this polyphagous species (Clarke 2016). However, studies in *D. melanogaster* suggest functional redundancy among OBPs, where multiple proteins bind the same or similar compounds, implying that a larger repertoire may enhance specificity rather than broaden the range of targets (Larter et al. 2016, Xiao et al. 2019). OBPs are also involved in reproduction, metabolism, and chemical resistance, with expression extending beyond chemosensory tissues (Pelosi et al. 2018, Rihani et al. 2021). These findings highlight how OBP expansions may have influenced the ecology and physiology of *C. capitata.*

Accurately identifying multigene families such as OBPs depends not only on the availability of sequence data but also on the quality of genome assemblies, which can fragment single genes, and on the depth of manual curation, both of which can lead to under- or overestimates of gene family size (Denton et al. 2014). For example, differences in the number and distribution of OBPs between the Ccap_2.1 and EGII-3.2.1 genomes reflect variations in assembly and annotation, which can result in differences in gene content due to methodological and annotation strategies (Denton et al. 2014).

In this case, although Ccap_2.1 RefSeq genome is more fragmented, with 2,354 scaffolds, it is supported by an advanced level of curation. It includes 14,236 genes, of which 12,563 encode proteins with high annotation quality, as evidenced by a BUSCO analysis reporting 99.4% completeness (Papanicolaou et al. 2016). In contrast, the EGII-3.2.1 genome, generated using more recent sequencing technologies, shows greater structural continuity (71 scaffolds) but lacks equivalent curation, as evidenced by the absence of annotated OBPs (Sollazzo et al. 2023). These contrasts illustrate how sequencing platforms and annotation depth influence gene resolution and functional annotation (Pavlidis et al. 2012, Feldmeyer et al. 2024).

RT-PCR analysis supported these findings by successfully amplifying 21 *Obp*s from the head tissues of sexually mature F_1_ wild-type males. Of these, 14 Ccap*Obp*s were identified in both genomes, the transcriptome, and additional NCBI sequences, confirming consistency across data sources. However, 7 amplified Ccap*Obp*s (*19a-like*, 19d, 19d-like, 28a, 69a, *83a-like*, and *84a-like*) were absent from the EGII-3.2.1 genome but present in Ccap_2.1 and in the transcriptome, except for 19d, 69a, and *84a-like*, which were also undetected in the transcriptome. These results underscore the added value of incorporating transcriptomic data, which has been shown in other systems to reveal genes missing from genome assemblies or clarify ambiguous gene models (Denton et al. 2014, Chen et al. 2015, Yin et al. 2019). Nonetheless, the transcriptome was restricted to male head tissue, which may have biased OBP recovery toward genes expressed in sexually mature males while omitting those active in other tissues, developmental stages, or in females (Pelosi et al 2018). At the same time, the focus on male heads provides relevant information for the sterile insect technique context, since these OBPs are expressed in sexually mature males and represent valuable candidates for future functional studies (Guimarães et al. 2025).

Using the homologized CcapOBP repertoire, we examined their evolutionary relationships with OBPs from other Tephritidae species. Bayesian phylogenetic analysis of 48 CcapOBPs and 307 representative OBPs from 8 tephritid species revealed patterns of evolutionary diversification within this multigene family. The dataset included members of 3 Tephritidae tribes: Dacini (*B. dorsalis*, *B. oleae*, *Z. cucurbitae*, *Z. tau*), Toxotrypanini (*A. fraterculus*, *A. ludens*, *A. obliqua*), and Trypetini (*R. pomonella*). The resulting tree resolved 3 major clades: (i) a large group of classical OBPs; (ii) a clade combining classical, Minus-C, and atypical OBPs; and (iii) a distinct Plus-C OBP clade. This topology is consistent with prior findings in *D. melanogaster* (Hekmat-Scafe et al. 2002).

The OBP repertoire in *C. capitata* is comparable to other tephritid species, with a similar number of genes as *B. dorsalis* (49 OBPs) and more than *Bactrocera papayae* Drew and Hancock (35), *Bactrocera correcta* Bezzi (34), *B. oleae* (36), *Z. cucurbitae* (33), and *Z. tau* (33). In contrast, *Anastrepha* species have fewer OBPs (Segura-León et al. 2022). This variation may reflect differences in study depth and data curation, as well as species-specific adaptations related to host range and ecological niche. Notably, highly polyphagous species like *B. dorsalis* and *C. capitata* possess more OBPs (Auer et al. 2021, Zhou et al. 2021).

The OBP phylogeny across 9 tephritid species reveals evolutionary patterns consistent with previous systematic and molecular studies in Tephritidae (Virgilio et al. 2015, Congrains et al. 2021, He et al. 2024). Most CcapOBPs clustered with OBPs from Dacinae species (*Bactrocera*, *Zeugodacus*), suggesting close evolutionary ties and conservation of ancestral genes. However, some CcapOBPs (47b, 73a, 69a, 19b, 19a, 99b, and 99c) clustered with more distantly related Trypetinae species, including Toxotrypanini (*Anastrepha*) and Trypetini (*Rhagoletis*). This pattern may indicate the functional conservation of ancestral genes shared before the divergence of these tribes or may be associated with post-divergence diversification events (Fay and Wu 2000, Zhang 2003).

Clades such as OBP73a, OBP69a, OBP44a, and OBP99b, where *C. capitata* OBPs clustered with Trypetini orthologs, included representatives from all analyzed tephritid genera, supporting the evolutionary robustness of these relationships. Notably, OBP99b and OBP99c showed closer relationships with species from the Dacini tribe. This diversification suggests potential adaptations linked to speciation and diversification processes within this subfamily (Rihani et al. 2021, Zhao et al. 2024). In contrast, OBPs such as CcapOBP19b, CcapOBP47b, and CcapOBP99d were only matched by orthologs in *A. fraterculus* and *Z. tau*, possibly reflecting gaps in available genomic data.

Also, the atypical subfamily comprised sequences exclusively identified in *C. capitata*, *A. fraterculus*, *and Z. tau*, sharing orthology with *D. melanogaster* (Rondón et al. 2022). The phylogenetic analysis used available datasets, including RefSeq sequences for *Z. cucurbitae*, *B. dorsalis*, and *B. oleae*; NCBI genomes for *A. ludens* and *A. obliqua*; and curated sequences from NCBI and UniProtKB for *Z. tau*, *A. fraterculus*, and *R. pomonella*. Incomplete or inconsistent data curation may hinder the resolution of phylogenetic relationships in less-represented OBP clades, underscoring the need for standardized, integrated datasets to enhance evolutionary analyses in Tephritidae.

Despite current data limitations, our findings reveal species-specific evolutionary events within the Dacinae subfamily. Notably, duplications of OBP56h and the expansion of the OBP99c-like group were exclusive to *C. capitata* and *B. dorsalis*, 2 highly polyphagous species. These expansions may enhance detection of diverse volatiles or increase sensitivity to host-related cues (Ma et al. 2024). Additionally, CcapOBP50e-like did not cluster with known tephritid OBPs but shared structural features typical of the Plus-C subfamily, including a signal peptide and conserved cysteines, and showed 21% similarity to *D. melanogaster* (Pearson and Sierk 2005).

This study provides a solid framework for understanding OBP diversity and evolution in *C. capitata*. By integrating genomic and transcriptomic data, homologating with *D. melanogaster*, validating with RT-PCR, and comparing across species, we curated and standardized a repertoire of 48 CcapOBPs. This reclassification revealed key evolutionary patterns such as duplication, diversification, and expansion, indicating adaptive evolution within this gene family. It also highlighted limitations related to genome assembly differences and data gaps in underrepresented species, emphasizing the need to refine CcapOBP nomenclature through future chromosomal mapping.

These findings underscore the importance of studying OBPs in tephritids and the need for comprehensive revision and curation of genomic data to improve homologation across OBP and other chemosensory protein families. Incomplete or inconsistent genetic representation in current databases can lead to misclassification and inaccurate phylogenetic inferences. Advancing data integration will not only deepen our understanding of chemosensory system evolution in *C. capitata* and related species but also strengthen the basis for applied research. Future work should focus on functionally characterizing CcapOBPs through ligand-binding, structural, and behavioral assays. In silico approaches such as docking, molecular dynamics, and virtual screening offer promising tools to identify candidate semiochemicals and to support the development of innovative strategies for integrated pest management.

## Supplementary Material

ieaf088_Supplementary_Data
